# Supplementary material for: Spectro-temporal acoustical markers differentiate speech from song across cultures
Source: Nat Commun. 2024 Jun 6;15:4835. doi: 10.1038/s41467-024-49040-3 (PMC11156671; doi:10.1038/s41467-024-49040-3)
Supplement: Supplementary file 3 — Reporting Summary [file 41467_2024_49040_MOESM3_ESM.pdf]

nature portfolio

Corresponding author(s): Philippe Albouy

Last updated by author(s): 2024/04/10

Reporting Summary

Nature Portfolio wishes to improve the reproducibility of the work that we publish. This form provides structure for consistency and transparency in reporting. For further information on Nature Portfolio policies, see our Editorial Policies and the Editorial Policy Checklist.

Statistics

For all statistical analyses, confirm that the following items are present in the figure legend, table legend, main text, or Methods section.

n/a	Confirmed	
☐	☒	The exact sample size (*n*) for each experimental group/condition, given as a discrete number and unit of measurement
☐	☒	A statement on whether measurements were taken from distinct samples or whether the same sample was measured repeatedly
☐	☒	The statistical test(s) used AND whether they are one- or two-sided<br>*Only common tests should be described solely by name; describe more complex techniques in the Methods section.*
☐	☒	A description of all covariates tested
☐	☒	A description of any assumptions or corrections, such as tests of normality and adjustment for multiple comparisons
☐	☒	A full description of the statistical parameters including central tendency (e.g. means) or other basic estimates (e.g. regression coefficient) AND variation (e.g. standard deviation) or associated estimates of uncertainty (e.g. confidence intervals)
☐	☒	For null hypothesis testing, the test statistic (e.g. F, t, r) with confidence intervals, effect sizes, degrees of freedom and P value noted<br>*Give P values as exact values whenever suitable.*
☒	☐	For Bayesian analysis, information on the choice of priors and Markov chain Monte Carlo settings
☒	☐	For hierarchical and complex designs, identification of the appropriate level for tests and full reporting of outcomes
☐	☒	Estimates of effect sizes (e.g. Cohen's *d*, Pearson's *r*), indicating how they were calculated

Our web collection on statistics for biologists contains articles on many of the points above.

Software and code

Policy information about availability of computer code

Data collection

Vocalization corpus

We used a corpus of 738 recordings of adult-directed song, and adult-directed speech from 6. People (N= 369) living in 21 societies produced each of these vocalizations, respectively, with a median of 15 individuals per society (range 6-57). From those for whom information was available, 86% were female.
Recordings were collected by the investigators of 6 and/or staff at their field sites, all using the same data collection protocol. They translated instructions to the native language of the participants, following the standard research practices at each site. Fieldsites were selected partly by convenience (i.e., via recruiting principal investigators at fieldsites) and partly to maximize cultural, linguistic, and geographic diversity (see Table S1).
For speech recordings, participants spoke to the researcher about a topic of their choice (e.g., they described their daily routine). For song, participants sang a song that was not intended for infants (see (6, for details); they also stated what that song was intended for (e.g., “a celebration song”). Participants vocalized in the primary language of their fieldsite, with a few exceptions (e.g., when singing songs without words; or in locations that used multiple languages, such as Turku, which included both Finnish and Swedish speakers).
Participants were free to determine the content of their vocalizations. This was intentional: imposing a specific content category on their vocalizations would likely alter the acoustic features of their vocalizations, which are known to be influenced by experimental contexts6.
All recordings were made with Zoom H2n digital audio recorders, using foam windscreens (where available). To ensure that participants were audible along with researchers, who stated information about the participant and environment before and after the vocalizations, recordings were made with a 360° dual x-y microphone pattern. This produced two uncompressed stereo audio files (WAV) per participant at 44.1 kHz; we only analyzed audio from the two-channel file on which the participant was loudest.
The investigator at each fieldsite provided standardized background data on the behavior and cultural practices of the society (e.g., whether there was access to mobile-phones/TV/radio, and how commonly people used ID speech or song in their daily lives). Most items were based

on variables included in the D-PLACE cross-cultural corpus6. The 21 societies varied widely in their characteristics, from cities with millions of residents (Beijing) to small-scale hunter-gatherer groups of as few as 35 people (Hadza). All of the small-scale societies studied had limited access to TV, radio, and the internet, mitigating against the influence of exposure to the music of other societies. Four of the small-scale societies (Nyangatom, Toposa, Sápara/Achuar, and Mbendjele) were completely without access to these communication technologies.

Behavioral experiment

Participants: 80 adults participated in the behavioral experiment. No statistical method was used to predetermine sample size. The group was composed of 80 native French speakers from France and Canada (33 female, 4 non-binary, mean age = 32.4 years +-10.86). Some of them (10 out of 80) were musically trained (more than 5 years of formal musical training). Six participants did not complete the entire test and the data of 74 participants were included in the current study. Participants reported no history of neurological or psychiatric disease. All participants provided written informed consent, and the experimental procedures were approved by the Ethics Review Board of the CIUSSS de la Capitale Nationale (2022-2476). The study has been conducted according to the principles expressed in the Declaration of Helsinki.
Procedure: The experiments took place in a sound-attenuated booth. Auditory stimuli were presented binaurally via Sennheiser HD 280 pro headphones at a comfortable sound level (~75 dB SPL). PsychoPy 57 was used to control the stimulus presentation and record responses. We played the song and speech recordings to these individuals who were asked to rate, as rapidly as possible on a 5-point scale on their keyboard, whether each speaker was singing (code 1) or speaking (code - 1). Participants had 9 seconds to respond and received no feedback (i.e., we did not tell them whether or not their rating was accurate). We did not provide any criteria to the listeners; they judged the sounds based on whatever they thought was relevant (hence the minimal instructions) so as to avoid any kind of bias about what features to use, hence providing a clean test of whether they would spontaneously use similar features as the classifier did. The experiment lasted approximately 15 minutes. We used 3 different blocks that were pseudo-randomly presented to the participant. Each bloc contained the same number of examples of speech and song for each society, with a total of 246 trials per block. This way a given listener was also rating the vocalization of the same speaker. Example of this task can be found online: https://run.pavlovia.org/palbouy/spectrotemp_bloc1.

Data analysis

MATLAB code are freely available at the following URL: https://osf.io/xcsqm/ 58

Extraction of spectro-temporal modulations
For the 738 selected samples (369 speech and 369 song) we decomposed the acoustical signal using the framework of spectrotemporal modulation power39. This analysis was using the duration of the shorter sample (song or speech) produced by the same speaker. The modulation domain results from the 2D fast Fourier transform of the autocorrelation matrix of the sound stimulus in its spectrographic representation and represents the energy modulation across the temporal and spectral axes (Fig. 1). This results in 738 STM patterns data that were then used for univariate and multivariate analyses.

Univariate analyses

Fieldtrip 40 functions as implemented in Brainstorm 41 were used to perform non-parametric permutation statistics with FDR correction (p <.001) for the contrast between song and speech STM patterns. Non-parametric tests were chosen as we did not make any assumption about the distribution of the STM data.

Multivariate analyses

Multivariate analyses were performed using MATLAB and linear support vector machine (SVM) implementation (https://www.mathworks.com/help/stats/fitcecoc.html). A linear classifier was chosen as STM data contains many more features than examples, and classification of such data is generally susceptible to over-fitting. One way of alleviating the danger of over-fitting is to choose a simple function (such as a linear function) for classification, where each feature affects the prediction solely via its weight and without interaction with other features (rather than more complex classifiers, such as nonlinear SVMs or artificial neural networks, which can let interactions between features and nonlinear functions thereof drive the prediction). With small stimulus sets it is typically necessary to regularize SVM analyses. The regularization parameter (□) serves as a degree of importance that is given to misclassifications. SVM pose a quadratic optimization problem that looks for maximizing the margin between both classes and minimizing the amount of misclassifications. Different values of □ will vary the misclassification constraint: when □ tends to infinite the solution tends to the hard-margin (allow no miss-classification). When □ tends to 0 the more the miss-classifications are allowed. Here the regularization parameter has been set to □ = 0.01 and has been selected using a separate validation set (infant directed song ang speech from 6, stimuli available here: https://zenodo.org/record/5525161). We performed the same analysis as reported above using SVM classifier with fieldsite-wise k-fold cross-validation to classify infant directed song and speech vocalization samples, using the STM as features. Results were also expressed as accuracy of category identification that was calculated using an average of the cross-validation folds. The selection of □ was done as follows: on the training set, we estimate several different models, with different values of the regularization parameter (□ = 0.1, □ = 0.05, □ = 0.01, □ = 0.005, □ = 0.001), then on the validation set, we choose the best model (the regularization parameter which gives the highest accuracy on the validation set).
Our strategy was to use the SVM classifier with fieldsite-wise k-fold cross-validation to classify song and speech vocalization samples, using the STM as features. The model is trained only on data from 20 of the 21 societies to predict whether each vocalization in the 21st society is song or speech. The procedure is repeated 21 further times, with each society being held out, to estimate the classification performance across the full set of societies. Results were expressed as accuracy of category identification that was calculated using an average of the cross-validation folds. For each classifier, we extracted the features weights (zscore) to evaluate the relative contribution of each feature in the classification. This procedure was performed across societies (21), across countries (18), language families (16), world subregions (15) and regions (6).

Behavioral data analysis: Data were processed with MATLAB (The Mathworks), and statistical analyses were performed with Jamovi (https://www.jamovi.org). For each participant, the ratings corresponding to scores in a linear scale (singing (code 1) to speaking (code -1), see Fig. 4) were extracted and averaged for each participant separately for each society, language family, countries, world subregions and world regions. These scores were analyzed with one sample t-tests and we performed Pearson's correlation between behavioral scores and decoding accuracy/raw spectro-temporal patterns that were corrected with FDR (p < 05) when necessary.

Analysis of acoustical data:

Acoustical analyses were done on a broad range of acoustical variables extracted in 6– and can be found here https://github.com/themusiclab/infant-speech-song/tree/main/data. All details about the extraction of these acoustical variables are described in the original article. These acoustical variables are summarized in the Table S2 (table from 6, used with permission).
We first aimed to investigate the link between acoustical features and STM data. To do so performed a Partial Least Squares (PLS) analysis as implemented in MATLAB https://www.mathworks.com/help/stats/plsregress.html using acoustical data as predictor variables and STM data as response variables. We used PLS instead of multiple linear regression models as multicollinearity existed between several variables of the acoustical dataset. PLS analysis was done for each spectral/temporal coordinate of the entire STM domain across all sounds (song and speech) resulting in one PLS model per spectral/temporal coordinate. For each model we then extracted the fitted responses values and estimated if the models could significantly fit the STM data. To do so, we computed the correlation (FDR corrected, p< .05) between the fitted responses (PLS models) and observed response (STM features) in the entire STM domain.
To determine which acoustical variables contributed more to the prediction, we calculated the variable importance in projection (VIP) scores for the PLS regression models for spectral and temporal statistical peaks reported in Figure 5 A. A VIP score is a measure of a variable's importance in the PLS model. In other words, it summarizes the contribution a variable makes to the model (see methods). The VIP score of a variable is calculated as a weighted sum of the squared correlations between the PLS components and the original variable. The weights correspond to the percentage variation explained by the PLS component in the model. As variables with a VIP score greater than 1 are considered important for the projection of the PLS regression model 42, we report in the main text only variables that were above this threshold. To facilitate illustration, we grouped variables according to their labels (see Table S2 for the corresponding labels) but present the complete list in Figs. S4 and S5).
Moreover, to investigate whether spectro-temporal modulation features were superior to classical acoustical features to differentiate speech and song vocalization, we used SVM classifier with fieldsite-wise k-fold cross-validation to classify song and speech vocalization samples, using either 1) only the STM features, 2) STM features and acoustical data, 3) acoustical data only or 4) acoustical data only but without the VIP variables as input features and compared model accuracy using RM ANOVA.
Finally, to investigated whether human judgments were linked to the acoustical features we computed Pearson's correlation between SVM decoding accuracy of the model using acoustical data only as features and the normalized difference between Song and Speech behavioral ratings computed within each society.

For manuscripts utilizing custom algorithms or software that are central to the research but not yet described in published literature, software must be made available to editors and reviewers. We strongly encourage code deposition in a community repository (e.g. GitHub). See the Nature Portfolio guidelines for submitting code & software for further information.

Data

Policy information about availability of data

All manuscripts must include a data availability statement. This statement should provide the following information, where applicable:

- Accession codes, unique identifiers, or web links for publicly available datasets
- A description of any restrictions on data availability
- For clinical datasets or third party data, please ensure that the statement adheres to our policy

Data availability: The data generated in this study have been deposited in the OSF database. Raw vocalizations and acoustical data are freely available at https://zenodo.org/record/5525161 and https://github.com/themusiclab/infant-speech-song/tree/main/data {Hilton, 2022 #6. The processed data are available at: DOI 10.17605/OSF.IO/XCSQM. Example of the judgment task can be found here: : https://run.pavlovia.org/palbouy/spectrotemp_bloc1

Research involving human participants, their data, or biological material

Policy information about studies with human participants or human data. See also policy information about sex, gender (identity/presentation), and sexual orientation and race, ethnicity and racism.

Reporting on sex and gender	*80 adults participated in the behavioral experiment. The group was composed of 80 native French speakers (Canadian and French, 33 female, 4 non-binary, mean age = 32.4 years +/- 10.86, self reported).*
Reporting on race, ethnicity, or other socially relevant groupings	*Race, ethnicity, or other socially relevant groupings were not considered in the behavioral experiment of the present study. We just note that all participants in the behavioral experiment were French speaking individuals, French or Canadian, and were thus familiar with western music*
Population characteristics	*mean age = 32.4 years +/- 10.86. Some of them (10 out of 80) were musically trained (more than 5 years of formal musical training). Participants reported no history of neurological or psychiatric disease.*
Recruitment	*Participants were recruited using social media, we did not identify any bias in during the recruitment*
Ethics oversight	Ethical approval was obtained from the Ethics Review Board of the CIUSSS de la Capitale Nationale (2022-2476). Participants provide informed consent.

Note that full information on the approval of the study protocol must also be provided in the manuscript.

Field-specific reporting

Please select the one below that is the best fit for your research. If you are not sure, read the appropriate sections before making your selection.

☐ Life sciences ☒ Behavioural & social sciences ☐ Ecological, evolutionary & environmental sciences

For a reference copy of the document with all sections, see nature.com/documents/nr-reporting-summary-flat.pdf

Life sciences study design

All studies must disclose on these points even when the disclosure is negative.

Sample size	NA
Data exclusions	NA
Replication	NA
Randomization	NA
Blinding	NA

Behavioural & social sciences study design

All studies must disclose on these points even when the disclosure is negative.

Study description	This study includes quantitative data. We first decomposed the acoustical signal of the vocalization samples using the Spectro-Temporal Modulation (STM) framework. We then contrasted the spectro-temporal modulation patterns of song and speech. vocalizations. We then used a Support Vector Machine (SVM) classifier with fieldsite-wise k-fold cross-validation to classify song and speech vocalization samples, using only the STM patterns as input features. We then studied naïve listeners' sensitivity to these spectro-temporal features. We played the song and speech recordings to 80 individuals who were asked to rank, as rapidly as possible on a 5-point scale, whether each speaker was singing (code 1) or speaking (code -1).<br>To test whether these listeners were using the spectro-temporal cues that distinguished song from speech in the prior analyses, we tested if the features identified on the STM patterns could predict their behavioral ratings. Finally, we studied whether spectro-temporal modulation features constitute a fundamental, and sufficient difference to account for how speech and song differ from one another, or whether acoustic features of the vocalizations might account for the results just as well. To do so, we used a broad range of acoustical variables extracted in 6(such as pitch (f0), first formant (f1), amplitude (intensity), pitch space, vowel rate, vowel space, roughness etc.) to test whether these variables were: i) correlated with spectro-temporal features, ii) could decode speech and song vocalizations with similar, higher or lower accuracy than with STM features, and iii) whether decoding accuracy for acoustical data can predict the behavioral scores of naïve listeners.
Research sample	80 adults participated in the behavioral experiment. No statistical method was used to predetermine sample size, but the number of participants in the present study in larger than in many psychoacoustical studies in the domain. The group was composed of 80 native French speakers from France and Canada (33 female, 4 non-binary, mean age = 32.4 years +-10.86). Some of them (10 out of 80) were musically trained (more than 5 years of formal musical training). Six participants did not complete the entire test and the data of 74 participants were included in the current study. Participants reported no history of neurological or psychiatric disease. All participants provided written informed consent, and the experimental procedures were approved by the Ethics Review Board of the CIUSSS de la Capitale Nationale (2022-2476). The study has been conducted according to the principles expressed in the Declaration of Helsinki.
Sampling strategy	Sampling procedure was random. No sample size calculation was performed but the number of participants in the present study in larger than in many psychoacoustical studies in the domain. Moreover, all effect sizes presented in the present study are very large, confirming that the sample size is appropriate. Data saturation has not been considered in the present study.
Data collection	The experiments took place in a sound-attenuated booth, no one was present in the room besides the participant and the researcher The researcher was aware of the study design and conditions. Auditory stimuli were presented binaurally via Sennheiser HD 280 pro headphones at a comfortable sound level (~75 dB SPL). PsychoPy was used to control the stimulus presentation and record responses. We played the song and speech recordings to these individuals who were asked to rate, as rapidly as possible on a 5-point scale on their keyboard, whether each speaker was singing (code 1) or speaking (code - 1). Participants had 9 seconds to respond and received no feedback (i.e., we did not tell them whether or not their rating was accurate).
Timing	*Start date November 2022 - end date January 2023*
Data exclusions	ix participants did not complete the entire test and the data of 74 participants were included in the current study.
Non-participation	No participant declined - 6 participants dropped out
Randomization	We used 3 different blocks that were pseudo-randomly (to have an equal number of participant per group) presented to the participant. Each bloc contained the same number of examples of speech and song for each society, with a total of 246 trials per

block. This way a given listener was also rating the vocalization of the same speaker. Example of this task can be found online: https://run.pavlovia.org/palbouy/spectrotemp_bloc1

Ecological, evolutionary & environmental sciences study design

All studies must disclose on these points even when the disclosure is negative.

Study description	NA
Research sample	NA
Sampling strategy	NA
Data collection	NA
Timing and spatial scale	NA
Data exclusions	NA
Reproducibility	NA
Randomization	NA
Blinding	NA

Did the study involve field work? ☐ Yes ☐ No

Field work, collection and transport

Field conditions	NA
Location	NA
Access & import/export	NA
Disturbance	NA

Reporting for specific materials, systems and methods

We require information from authors about some types of materials, experimental systems and methods used in many studies. Here, indicate whether each material, system or method listed is relevant to your study. If you are not sure if a list item applies to your research, read the appropriate section before selecting a response.

Materials & experimental systems

n/a	Involved in the study	
☒	☐	Antibodies
☒	☐	Eukaryotic cell lines
☒	☐	Palaeontology and archaeology
☒	☐	Animals and other organisms
☒	☐	Clinical data
☒	☐	Dual use research of concern
☒	☐	Plants

Methods

n/a	Involved in the study	
☒	☐	ChIP-seq
☒	☐	Flow cytometry
☒	☐	MRI-based neuroimaging

Antibodies

Antibodies used	NA
Validation	NA

Eukaryotic cell lines

Policy information about cell lines and Sex and Gender in Research

Cell line source(s)	NA
Authentication	NA
Mycoplasma contamination	NA
Commonly misidentified lines (See ICLAC register)	NA

Palaeontology and Archaeology

Specimen provenance	NA
Specimen deposition	NA
Dating methods	NA

☐ Tick this box to confirm that the raw and calibrated dates are available in the paper or in Supplementary Information.

Ethics oversight	NA

Note that full information on the approval of the study protocol must also be provided in the manuscript.

Animals and other research organisms

Policy information about studies involving animals; ARRIVE guidelines recommended for reporting animal research, and Sex and Gender in Research

Laboratory animals	NA
Wild animals	NA
Reporting on sex	NA
Field-collected samples	NA
Ethics oversight	NA

Note that full information on the approval of the study protocol must also be provided in the manuscript.

Clinical data

Policy information about clinical studies

All manuscripts should comply with the ICMJE guidelines for publication of clinical research and a completed CONSORT checklist must be included with all submissions.

Clinical trial registration	NA
Study protocol	NA
Data collection	NA
Outcomes	NA

Dual use research of concern

Policy information about dual use research of concern

Hazards

Could the accidental, deliberate or reckless misuse of agents or technologies generated in the work, or the application of information presented in the manuscript, pose a threat to:

No	Yes	
☒	☐	Public health
☒	☐	National security
☒	☐	Crops and/or livestock
☒	☐	Ecosystems
☒	☐	Any other significant area

Experiments of concern

Does the work involve any of these experiments of concern:

No	Yes	
☒	☐	Demonstrate how to render a vaccine ineffective
☒	☐	Confer resistance to therapeutically useful antibiotics or antiviral agents
☒	☐	Enhance the virulence of a pathogen or render a nonpathogen virulent
☒	☐	Increase transmissibility of a pathogen
☒	☐	Alter the host range of a pathogen
☒	☐	Enable evasion of diagnostic/detection modalities
☒	☐	Enable the weaponization of a biological agent or toxin
☒	☐	Any other potentially harmful combination of experiments and agents

Plants

Seed stocks	NA
Novel plant genotypes	NA
Authentication	NA

ChIP-seq

Data deposition

☐ Confirm that both raw and final processed data have been deposited in a public database such as GEO.

☐ Confirm that you have deposited or provided access to graph files (e.g. BED files) for the called peaks.

Data access links<br>*May remain private before publication.*	NA
Files in database submission	NA
Genome browser session<br>(e.g. UCSC)	NA

Methodology

Replicates	NA
Sequencing depth	NA
Antibodies	NA
Peak calling parameters	NA
Data quality	NA
Software	NA

Flow Cytometry

Plots

Confirm that:

☐ The axis labels state the marker and fluorochrome used (e.g. CD4-FITC).

☐ The axis scales are clearly visible. Include numbers along axes only for bottom left plot of group (a 'group' is an analysis of identical markers).

☐ All plots are contour plots with outliers or pseudocolor plots.

☐ A numerical value for number of cells or percentage (with statistics) is provided.

Methodology

Sample preparation	NA
Instrument	NA
Software	NA
Cell population abundance	NA
Gating strategy	NA

☐ Tick this box to confirm that a figure exemplifying the gating strategy is provided in the Supplementary Information.

Magnetic resonance imaging

Experimental design

Design type	NA
Design specifications	NA
Behavioral performance measures	NA

Acquisition

Imaging type(s)	NA
Field strength	NA
Sequence & imaging parameters	NA
Area of acquisition	NA

Diffusion MRI ☐ Used ☐ Not used

Preprocessing

Preprocessing software	NA
Normalization	NA
Normalization template	NA
Noise and artifact removal	NA
Volume censoring	NA

Statistical modeling & inference

Model type and settings	NA
Effect(s) tested	NA

Specify type of analysis: ☐ Whole brain ☐ ROI-based ☐ Both

Statistic type for inference<br>(See Eklund et al. 2016)	NA
Correction	NA

Models & analysis

n/a	Involved in the study
☒	☐ Functional and/or effective connectivity
☒	☐ Graph analysis
☒	☐ Multivariate modeling or predictive analysis
